# Assessment of the Effectiveness of Protective Coatings in Preventing Steel Corrosion in the Marine Environment

**DOI:** 10.3390/polym17030378

**Published:** 2025-01-30

**Authors:** Nicoleta Bogatu, Daniela Laura Buruiana, Alina Crina Muresan, Viorica Ghisman, Anca Lupu, Laurentiu Mardare, Elena Emanuela Herbei, Vasile Basliu, Alina Ceoromila, Stefan Florescu

**Affiliations:** 1 Interdisciplinary Research Centre in the Field of Eco-Nano Technology and Advance Materials CC-ITI, Faculty of Engineering, “Dunarea de Jos” University of Galati, 47 Domneasca Street, 800008 Galati, Romania; nicoleta.simionescu@ugal.ro (N.B.); alina.muresan@ugal.ro (A.C.M.); viorica.ghisman@ugal.ro (V.G.); elena.herbei@ugal.ro (E.E.H.); 2 Faculty of Medicine and Pharmacy, Dunarea de Jos University of Galati, 35 Al. I. Cuza Street, 800010 Galati, Romania; 3 Amrared Research Consulting S.R.L., 54 Brailei Street, 800090 Galati, Romania; laurentiu.mardare@ugal.ro; 4 Cross-Border Faculty, Dunarea de Jos University of Galati, 47 Domneasca Street, 800201 Galati, Romania; vasile.basliu@ugal.ro (V.B.); alina.cantaragiu@ugal.ro (A.C.); 5 National Institute of Marine Geology and Geo-Ecology (GeoEcoMar), 23-25 Dimitrie Onciul St., 024053 Bucharest, Romania; stefan.florescu@geoecomar.ro

**Keywords:** corrosion resistance, electrochemical methods, protective coatings, seawater, effect of time

## Abstract

This research aims to evaluate the effectiveness of protective coatings in preventing the corrosion of steel in the marine environment. Electrochemical tests were performed on S355JR steel immersed in natural seawater (Black Sea, Port Constanta) over a period of 22 weeks, using electrochemical techniques such as the evolution of the open circuit potential (OCP) and linear polarization resistance to calculate R_p_ and the corrosion rate (V_corr_). The investigated steel surfaces included (a) S355JR steel blasted with Al_2_O_3_, (b) S355JR steel blasted and coated with epoxy primer enriched with zinc, (c) S355JR steel blasted and coated with epoxy primer and polyurethane paint, and (d) S355JR steel blasted and subsequently coated with epoxy primer and then polyurethane paint to which kreutzonit particles had been added. The proportion of kreutzonit particles added to the polyurethane paint was 2 wt% of the total mass of the paint. Subsequently, the samples were subjected to morphological analyses and cross-sectional analysis by scanning electron microscopy (SEM), topographical characterization (roughness and microhardness), and structural assessments (FTIR and XRD), as well as an analysis of hydrophobicity (contact angle). The results of this study revealed significant differences in corrosion behavior between the different surfaces and coatings tested. Electrochemical analysis revealed that the coating with epoxy primer and polyurethane paint to which kreutzonit particles had been added provided the best corrosion protection in the marine environment during immersion.

## 1. Introduction

Corrosion is a major issue in modern society, causing annual losses of billions of dollars [1,2,3]. These include direct costs from material damage and indirect costs such as labor, equipment for corrosion control, income loss due to product degradation, and reduced operational safety [1,2,3]. About 90% of corrosion losses affect ferrous materials used in bridges, reinforcements, vehicles, agricultural equipment, ships, and port facilities [1,2,3,4].

Marine corrosion is a permanent problem, not only for moving and stationary ships but also for all port installations, desalination installations, and objects that come into prolonged contact with water and the marine atmosphere [5,6,7]. The corrosivity of seawater depends on many factors, such as salinity, dissolved oxygen content, temperature, biological factors, the relative movement of the corrosive medium relative to the material subject to corrosion, etc. Depending on the metallic material subjected to the action of seawater, all these factors can also determine the type of corrosion that can occur [5,6,7]. S355JR steel is a type of high-strength structural steel used in various applications, including marine metal structures [8]. However, in marine environments, corrosion is a major concern because salt water and humid air can accelerate the deterioration process of metals [5,6,7]. In this context, functional surfaces play a crucial role in protecting marine metallic structures [2]. Corrosion over time affects steels, as they are susceptible to rust formation in the presence of humidity and corrosive substances commonly found in the marine environment [9]. Corrosion can weaken metal structures and lead to a decrease in their lifespan [9]. In order to prevent or slow down corrosion in marine environments, various strategies have been developed over time, such as polymeric anticorrosion coatings [10,11,12,13,14,15], ceramic coatings [16], electrodeposition of nanocrystalline materials [17], and self-assembled nanocoatings [18].

Research in recent years has given increased attention to the use of polymeric coatings for anticorrosion protection, in which different types of micro/nanoparticles have been dispersed [11,12,19,20,21,22,23,24,25,26,27,28,29,30,31,32]. These microparticles/nanoparticles can act as corrosion inhibitors, releasing protective chemicals during corrosive attack, thus preventing steel damage [21,22]. Anticorrosive polymer coatings provide a physical barrier against moisture and corrosive substances, thereby preventing their contact with the metal surface [10,11,12,13,14,33,34]. This study aims to evaluate the corrosion resistance of S355JR steel in natural seawater over 22 weeks using electrochemical methods, considering different surface treatments: (a) S355JR steel blasted with Al_2_O_3_, (b) S355JR steel blasted and coated with epoxy primer, (c) S355JR steel blasted and coated with epoxy primer and polyurethane paint, and (d) S355JR steel blasted and subsequently coated with epoxy primer and then polyurethane paint to which kreutzonit particles had been added. No studies have been reported in the literature on the use of kreutzonit particles in polymer coatings to enhance the corrosion resistance of S355JR steel immersed in natural seawater for 22 weeks. Additionally, there are few long-term studies in the literature evaluating the effectiveness and durability of anticorrosion coatings used in the maritime industry, particularly those tested in natural seawater, where corrosion factors such as salts can accelerate material degradation.

According to information provided by the manufacturer, kreutzonit particles were selected for this study due to their exceptional chemical stability, mechanical strength, and insulating properties, primarily provided by their high ZrO_2_ content. This composition forms a dense and impermeable barrier that effectively protects the substrate from corrosive agents. Kreutzonit particles also offer excellent thermal and mechanical stability, ensuring strong adhesion and long-term performance, especially in marine environments. Furthermore, their use is more cost-effective compared to nanometer-sized particles, making them an efficient and economical solution for improving corrosion resistance. This addition aims to provide additional protection against corrosion factors and optimize the performance of the corrosion protection system in the marine environment.

The results obtained in this study can serve as a guide for the appropriate selection and application of protective coatings in the marine industry and other areas where corrosion in the marine environment is a significant problem.

## 2. Materials and Methods

### 2.1. Materials

S355JR steel (chemical composition provided in Table 1), purchased from Mairon Galati, Romania, in the form of 500 × 500 × 2 mm plates, was used as the substrate for polymer coatings. For electrochemical experiments, the samples were cut to 25 × 25 × 2 mm dimensions. After cutting, the samples were sandblasted following the ISO 8501/1-88 standard at Sa 2½ cleaning grade [35]. Sandblasting was performed using alumina (Al_2_O_3_) abrasive sand, provided by Abraziv Trade SRL, Mureș, Romania. The technical data of the abrasive sand, as well as the equipment and parameters used, were described in a previous study [36].

To remove excess dust from the samples resulting from the sandblasting process, the samples were blown with compressed air. After the S355JR steel samples were blasted and blown with compressed air, they were cleaned with acetone before the coating process. The zinc-rich epoxy primer used in this study was a two-component product Zincamid G3202-1, and the hardener used was I 324. The polyurethane paint used was also a two-component product Polidur E3900, the hardener used was I 398, and the thinner was D391. After the S355JR steel samples were blasted, a zinc-rich epoxy primer was applied to the blasted surface using a metal roller, within two hours of completing the surface preparation. The mixing ratio of the zinc-rich epoxy primer (Zincamid G3202-1) to the hardener (I 324) was 5:1. After drying the epoxy primer coat at room temperature for 6 h, the polyurethane paint was applied over the samples. The mixing ratio for the two-component polyurethane paint was 6:1 between the main substance and the hardener, using thinner D391 and hardener I 398.

The micrometric kreutzonit powder used in this study was purchased from Interceram, Sighisoara, Romania. The physical and chemical properties of the kreutzonit powder, as provided by the supplier, are presented in Table 2.

**Figure 1:**
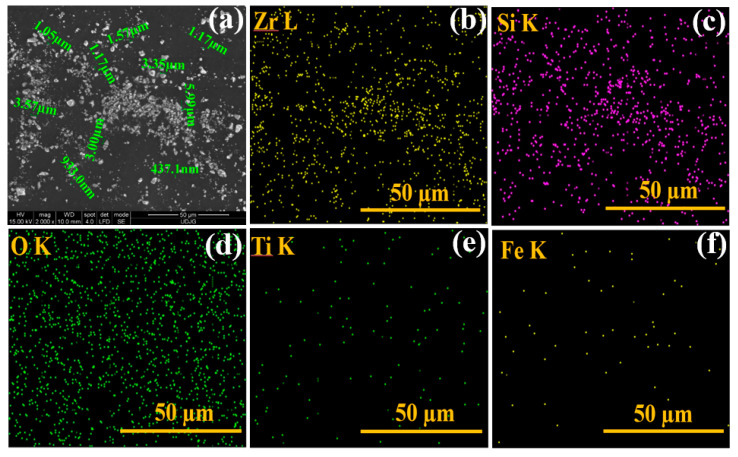
SEM morphology of kreutzonit microparticles (**a**); EDX elemental map (**b**–**f**) including Zr L, Si K, O K, Ti K, and Fe K.

In addition to the technical information provided by the supplier, morphological and elemental analyses of the kreutzonit powder were performed and the results are presented in Figure 1. The kreutzonit powder was dispersed in an ethyl alcohol solution using an ultrasound bath for 10 min. The resulting solution was then applied dropwise onto a circular pin with a carbon strip attached, using a pipette.

From Figure 1, it can be seen that the kreutzonit powder is presented in the form of agglomerations on the carbon strip and that the particles used have micrometric dimensions.

### 2.2. Preparation of Microcomposite Polymeric Layer by Adding Kreutzonit Particles

In the composition of polyurethane paint, kreutzonit microparticles were introduced, representing approximately 2 wt% of the total mass of the paint through the sonication process.

In accordance with the research of other authors [21,37,38,39], the sonication was performed at room temperature for 2 h with the help of a Hielscher UP400St ultrasonic processor at an amplitude of 40% and 0.5 cycles per second on/off. To avoid temperature rise during sonication, cooling of the mixture was achieved by immersing the dish in a mixture of water and ice [39].

Afterwards, the hardener was carefully added manually at a 1:6 mix ratio of hardener to polyurethane resin. After this process, the protective layer with the addition of kreutzonit microparticles was applied over the S355JR samples sandblasted and covered with epoxy primer with the help of a metal roller to avoid the formation of gaps/air bubbles and the removal of excess paint. For each type of material/coating studied, 50 sets of samples were prepared.

Table 3 shows the abbreviations adopted in this study. This coating system for naval steel (zinc-enriched epoxy primer and polyurethane paint) was chosen because data from the specialized literature demonstrate that this combination provides the best protection against corrosion [40].

The zinc-enriched epoxy coating system provides excellent cathodic protection, preventing rust and extending the steel’s durability [41], and the polyurethane paint applied over the epoxy primer adds increases UV resistance and flexibility, preventing deterioration and cracking under temperature variations and mechanical stress [40].

Studies and research in the field confirm the effectiveness of this combination in the aggressive marine environment, providing long-term corrosion protection and maintaining the structural integrity of the steel [40,41]. In addition, the novelty of the study consists of the addition of kreutzonit microparticles in the polyurethane paint, which considerably improves this system, offering additional protection against corrosion.

### 2.3. Electrochemical Mesurements

Electrochemical studies on untreated and treated S355JR steel were performed using PGP 201 Potentiostat/Galvanostat equipment, and data acquisition was carried out using VoltaMaster 4 version 7.10 software. The electrochemical cell used was a classical one composed of three electrodes (the working electrode (made of the studied material S355JR, both untreated and treated), the reference electrode (made of Ag/AgCl with saturated solution of KCl potential versus NHE = +199 mV), against which all measured potentials were recorded, and the counter electrode (a pure platinum electrode). To establish electrical contact, the working electrode (treated and untreated S355JR steel) was soldered to a copper wire, after which the samples were embedded in epoxy resin to form a well-defined and constant active surface of 3 cm^2^ ± 0.4 cm^2^. The electrolyte used for the electrochemical tests was natural seawater that was collected from Constanta Port with the help of the multifunctional marine research vessel “Mare Nigrum” from a depth of 3 m using the MN176-091 test station with the CTD system SBE 25, model SBE 32 rosette, and 5 l Niskin cylinders during the ascent to the surface.

**Figure 2:**
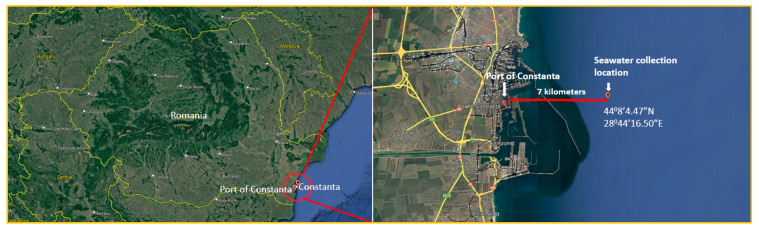
Seawater collection location map of Port Constanța, Romania (Google Earth Pro software 7.3.6.9345 version).

The coordinates of the testing station MN176-091 on board the multifunctional marine research vessel “Mare Nigrum” are presented in Figure 2.

The physical–chemical characteristics of the natural seawater used were measured using a multiparameter Phoenix EC-15 multi tester and are presented in Table 4.

The electrochemical tests were performed at ambient temperature over a period of 22 weeks and the volume of electrolyte used in the electrochemical cell was 200 mL. The times at which the samples were measured were T0 = at immersion; T1 = after 2 weeks; T2 = after 4 weeks; T3 = after 6 weeks; T4 = after 8 weeks; T5 = after 10 weeks; T6 = after 12 weeks; T7 = after 14 weeks; T8 = after 16 weeks; T9 = after 18 weeks; T10 = after 20 weeks; and T11 = after 22 weeks. The experimental protocol imposed at each monitoring time until the end of the 22 weeks was as follows:

- Measurement of the open circuit potential for 1 h, with a measuring period of 0.6s.
- General corrosion—*R_p_* and *V_corr_*; scan rate—5 mV/s; overvoltage—40 mV; OCP duration—1 min; determined measure—50 Rp; step duration—0.6 s; step amplitude—3 mV; total time—70 min; and initial scan—cathodic.

Each measurement sequence (*R_p_*-*V_corr_*) comprised 50 data points derived from an equal number of linear polarization curves using the Stern–Geary equation (Equations (1) and (2)) [36].

(1) $$i_{cor}=\frac{B}{R_{p}}$$

(2) $$B=\frac{b_{a}|b_{c}|}{2.303(b_{a}+|b_{c}|)}$$

where *B* = the specific constant; *R_p_* = polarization resistance; *i_cor_* = corrosion current density; and *b_a_* and *b_c_* are the Tafel slopes for the anodic and cathodic reactions.

For each sample, tests were performed at least three times to ensure the repeatability of measurements. Acquired data were interpreted and analyzed using Origin 2022 software.

### 2.4. Characterization of Surfaces Studied

The morphology of the uncoated and coated S355JR steel samples and thickness of the protection layers were determined by scanning electron microscopy (SEM) using FEI Quanta 200 (FEI Company, Hillsboro, OR, USA).

The elemental analysis of the uncoated and coated S355JR steel samples and mapping of images were performed using energy-dispersive X-ray (EDX) analysis.

In order to reduce charging effects before examination using SEM, the samples were covered with an 8 nm sputtered gold layer using an SPI-Module system (SPI Module™ Supplies, West Chester, PA, USA).

The structural analyses of the uncoated and coated S355JR steel samples were performed by Fourier transform infrared (FTIR) spectroscopy (IRSpirit-T FT-IR Spectrometer Shimadzu, Tokyo, Japan) using a built-in ATR accessory-type QATR-S, DLATGS detector (Shimadzu, Tokyo, Japan), and KBr beam splitter over the range of 4700–369 cm^−1^ at room temperature with a resolution of 2 cm^−1^ and a scan number of 45.

For the confirmation of crystallinity and phase formation of the sample, X-ray diffraction (XRD) measurements were taken on Dron-3 equipment (Bourevestnik Inc., St. Petersburg, Rusia) with Co Kα radiations (λ = 1.790300 Å). The X-ray diffractometer operated at a voltage of 30 kV and 20 mA current, with a step of 0.05°/s, a time exposure of 3 s, and a total time/sample of 2 h and 13 min, in a range 2θ = 15–90°.

The diffraction patterns obtained were analyzed using the software Match! 3 (using Rietveld refinement), and the reference database library used was the Crystallography Open Database (COD).

Two-dimensional roughness measurements of the samples studied were carried out using a Mitutoyo Surftest SJ-210 tester over a distance of 4 mm and with a speed of 0.20 μm/s.

Three determinations were made on each sample, after which the average value of the roughness parameter R_a_ was calculated.

The microhardness of the samples studied was measured using an Insize digital microhardness tester, which was equipped with a standard Vickers-type diamond indenter (indenter of 0.1 kgf/mm^2^). On each sample, a set of 3 measurements were taken (and the exposure period force for each measurement was 10 s).

Wettability was measured by the static sessile drop method on an Ossila Contact Angle goniometer equipped with a digital camera for recording, and OCA v.4.13.0 software was used for processing the results of the samples studied.

The samples were fixed with clamps on the test table of goniometer and very small water drops were deposited on the surface using the micro syringe (distilled water drop volume used = 3 μL).

The contact angle value was recorded after 5 s of distilled water–substrate contact. A set of 3 measurements were taken on each sample.

## 3. Results and Discussions

### 3.1. Coating Thickness

**Figure 3:**
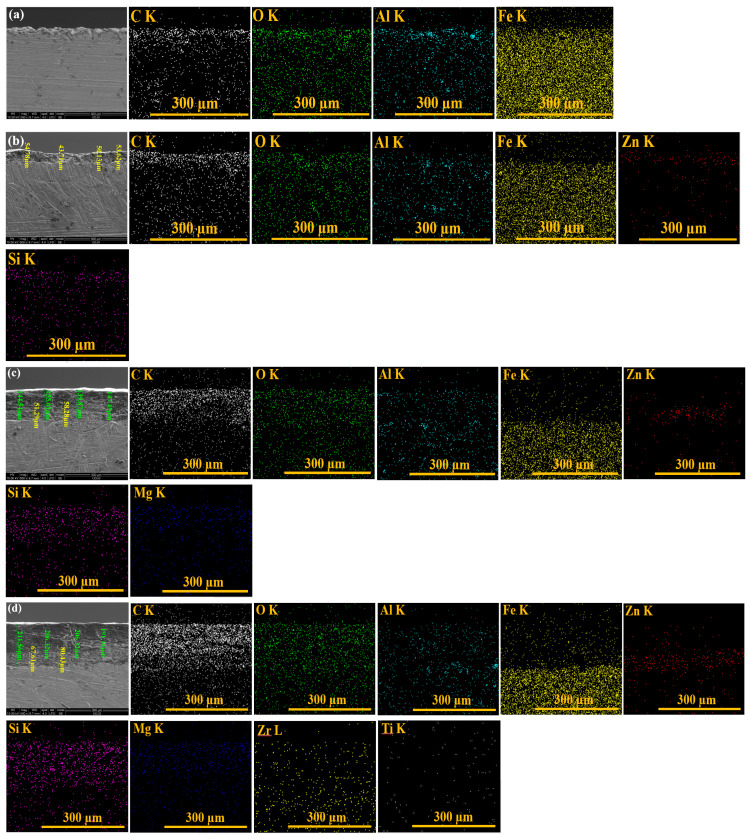
Cross-sectional SEM-EDX elemental map images of the coating thickness studied for (**a**) S355JR S, (**b**) S355JR SG, (**c**) S355JR SGV, and (**d**) S355JR SGVMp.

Figure 3 shows cross-sectional SEM-EDX elemental map images of the coating thickness studied for (a) S355JR S, (b) S355JR SG, (c) S355JR SGV, and (d) S355JR SGVMp.

From Figure 3a, it can be seen that the surface of the S355JR steel without the anticorrosion protection coating is uneven due to the Al_2_O_3_ sandblasting process.

The EDX analysis shows the presence of C, Fe, O, and Al (Al due to the sandblasting grit used). In Figure 3b, it can be seen that the surface of the S355JR SG has a layer thickness of approximately 51 µm.

From the distribution of the maps of the elements identified by EDX, the presence of Zn and Si can be observed in the uncoated sample.

For the sample of S355JR SGV, it can be seen that it has a total layer thickness of approximately 147 µm (Figure 3c). From the distribution of the elemental maps, the presence of Mg can be observed in the sample coated with epoxy primer.

The layer thickness measured for S355JR SGVMp has a value of approximately 204 µm (Figure 3d). The distribution of the element maps for this sample confirms the inclusion of kreutzonit particles in the polymer coating.

### 3.2. Contact Angle and Roughness of the Surface Studied

**Figure 4:**
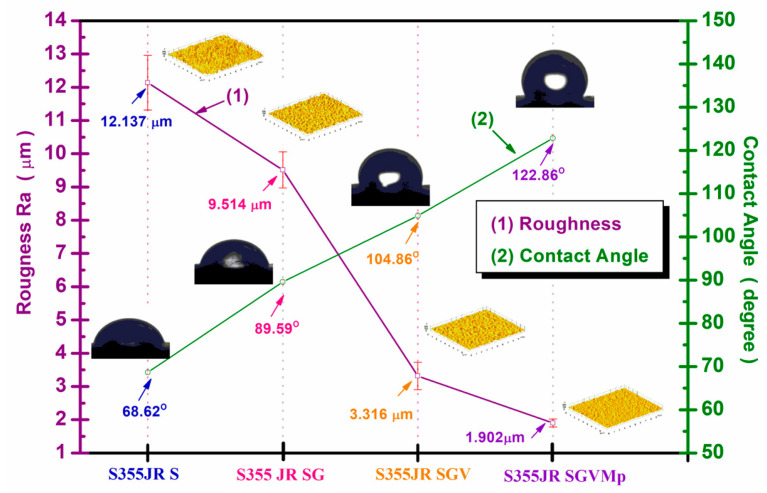
Contact angle and surface roughness of S355JR S; S355JR SG; S355JR SGV; and S355JR SGVMp.

Figure 4 shows the evolution of the contact angle and roughness for the studied surfaces. The 3D images of the roughness shown in Figure 4 were obtained with the help of the ImageJ software version 1.54i program by processing the SEM micrographs of the sample surfaces analyzed before corrosion, presented in Section 3.6.

From Figure 4, it can be seen that for the surface S355JR S, the roughness had the highest value, R_a_ = 12.137 ± 0.823 µm, and the contact angle had a value of 68.62 ± 0.52^0^. For the surface S355JR SG, it can be seen that the roughness value decreased compared to the sample without a protective coating to a value of R_a_ = 9.514 ± 0.541 µm, while the value of the contact angle increased to a value of 89.59 ± 0.60^0^ compared to the sample without a protective coating.

For the S355JR SGV sample, the roughness decreased to a value of 3.316 ± 0.415 µm and the contact angle increased to a value of 104.86 ± 0.11°. The S355JR SGVMp sample exhibited the lowest roughness value among all the samples analyzed in this study, measuring 1.902 ± 0.123 µm. Additionally, it showed the highest contact angle of 122.86 ± 0.80°.

This behavior can be explained by the fact that the microparticles of kreutzonit added to the polymer coating fill the voids and asperities, thus creating a smoother and more uniform surface. From Figure 4, it can be seen that the roughness and the contact angle are inversely proportional, i.e., the roughness decreased and the contact angle increased. Similar behavior has been observed in the literature by other authors [42].

Roughness and contact angle are two important aspects in corrosion protection coatings. It is known in the specialized literature that surface roughness plays a crucial role in influencing corrosion processes [36].

While rough surfaces can promote corrosion by providing more sites for initiation pitting corrosion, smoother surfaces are generally more resistant to corrosion due to their reduced tendency for pitting corrosion and increased compatibility with protective coatings [36,43].

Hydrophobicity is extremely important in anticorrosive protective coatings in the naval field, as it can help prevent corrosion, erosion, and degradation of coatings in an aggressive environment such as the marine one [44,45].

### 3.3. Microhardness of the Surface Studied

**Figure 5:**
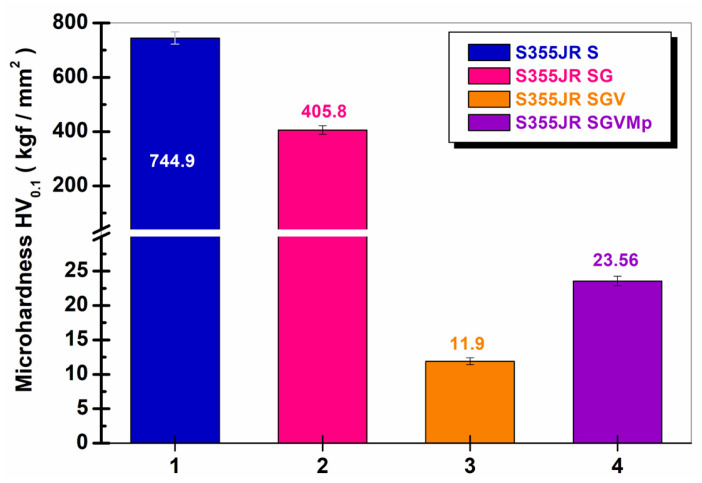
Microhardness of (1) S355JR S, (2) S355JR SG, (3) S355JR SGV, and (4) S355JR SGVMp.

Figure 5 shows the values obtained for the microhardness of the investigated steel surfaces.

From Figure 5, it can be seen that the Vickers microhardness for the sandblasted steel sample had a value of 744.9 ± 36.2 kgf/mm^2^, while for the sample of S355JR SG, the microhardness decreased to a value of 405.8 ± 15.6 kgf/mm^2^. For the sample of S355JR SGV, it was observed that the microhardness value decreased the most compared to the other samples to a value of 11.9 ± 0.23 kgf/mm^2^. For S355JR SGVMp, a slight increase in microhardness was observed compared to the S355JR SGV sample due to the addition of kreutzonit microparticles, with a value of 23.56 ± 0.91 kgf/mm^2^. Similar results were also observed in the literature by other authors [12].

The reduced microhardness values observed in samples coated with epoxy primer and polyurethane paint enriched with kreutzonit particles can be attributed to the intrinsic properties of the polymeric materials utilized in these protective layers [2,11,12,21,23].

Epoxy primers and polyurethane paints are characterized by flexible molecular structures with relatively weak intermolecular chemical bonds. This configuration results in lower mechanical strength and reduced intrinsic hardness compared to metallic or ceramic materials, rendering the coating more susceptible to deformation under mechanical stress [2,11,12,21,23].

However, as illustrated in Figure 5, samples incorporating kreutzonit microparticles exhibited a slight increase in microhardness compared to coatings without microparticle additives. This enhancement indicates that, despite the mechanical limitations of the polymer matrix, the inclusion of kreutzonit particles contributes to reinforcing the coating layer. The kreutzonit microparticles likely serve as localized reinforcement sites, mitigating deformation under applied loads and marginally improving the mechanical durability of the protective coating. Although this improvement is modest, it validates the positive impact of microparticle integration on the mechanical performance of the coating system.

### 3.4. Electrochemical Evaluation of the Coatings

#### 3.4.1. Variation in OCP (Open Circuit Potential) During Immersion Time

**Figure 6:**
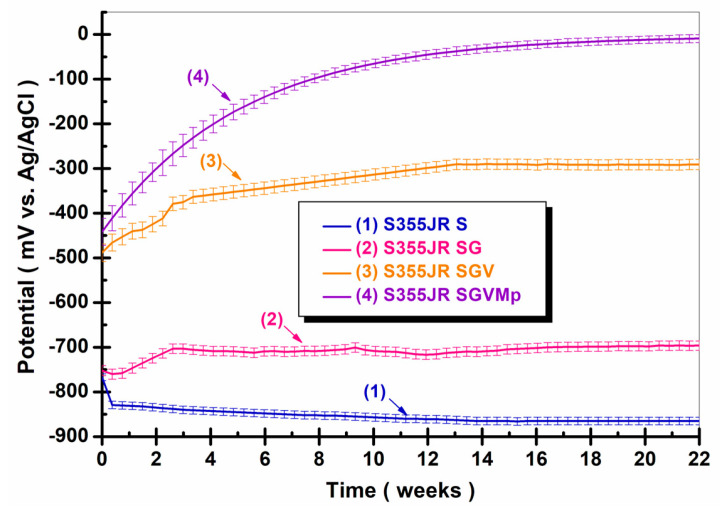
Variation in OCP during immersion time (22 weeks) in natural seawater for (1) S355JR S, (2) S355JR SG, (3) S355JR SGV, and (4) S355JR SGVMp.

Determining the evolution of the open circuit potential (presented in Figure 6) is a useful electrochemical method because monitoring this parameter (potential vs. time) gives us information about the potential changes of a material immersed in an electrochemical environment—changes that can indicate processes such as the removal of layers of oxide (initiation of corrosion), the formation of an oxide film, or a steady state (oxide neither dissolves nor forms) [11,12,46,47,48].

However, it is important to emphasize that the corrosion potential itself is not always a precise indicator of the degree of corrosion because it is a more qualitative than quantitative method [11,12,46,47,48].

From Figure 6, it can be seen that at the beginning of the immersion, S355JR S had a potential value of −767.02 ± 6.4 mV vs. Ag/AgCl and moved towards more negative values until the end of the immersion period, reaching a potential value of −865.04 ± 8.9 mV vs. Ag/AgCl. A shift in the potential towards more negative values in the case of sandblasted carbon steels was also observed in the specialized literature by other authors [11,12,36]. In general, a decrease in the values of open circuit potential can suggest a deterioration of the native oxide layer and the initiation of corrosion, while its increase can indicate the formation of a protective film on the surface of the material [11,12,46,47,48].

For S355JR SG, slight variations in the potential can be observed during the duration of immersion (22 weeks). At immersion, the sample covered with epoxy primer had a potential value of −752.25 ± 11.2 mV vs. Ag/AgCl; this value decreased in the first 3 days to a value of −762.27 ± 11.4 mV vs. Ag/AgCl. After this period, the potential value increased compared to the potential value from immersion, reaching a potential value of −696.26 ± 10.5 mV vs. Ag/AgCl at the end of the experiment.

The equilibrium state in the case of this sample was reached around the 16th week after immersion. Making a comparison between the sandblasted sample and the sample covered with epoxy primer, it can be observed that the value of the potential from the immersion and the value of the potential at the end of the immersion period were more positive for the sample coated with epoxy primer compared to the sandblasted sample without a protective coating. In the case of the epoxy coating over which polyurethane paint was added (S355JR SGV), it can be observed that the value of the potential was higher compared to the sample coated with primer and the sample without a protective coating. At immersion, it had a value of −488.37 ± 19.5 mV vs. Ag/AgCl, while at the end of the immersion period, it reached a potential value of −291.02 ± 11.6 mV vs. Ag/AgCl. The displacement of the potential in the case of S355JR SGV between the immersion period and the period from the end of the immersion was ΔE = 197.35 mV vs. Ag/AgCl. An increase in potential indicates the formation of a protective film on the surface of the material, implicitly indicating better corrosion resistance.

Making a comparison between the samples studied in Figure 6, it is obvious that the potential value reached the most positive values for the S355JR S sample, which was subsequently coated with epoxy primer and then polyurethane paint to which kreutzonit particles had been added, thus confirming the effectiveness of the anticorrosion protection coating to which kreutzonit particles were added compared to the S355JR S, S355JR SG, and S355JR SGV samples.

For S355JR SGVMp, the immersion potential had a value of −441.92 ± 30.1 mV vs. Ag/AgCl, reaching a value of −9.05 ± 4.9 mV vs. Ag/AgCl at the end of the experiment; the potential difference between immersion and the end of the experiment was ΔE = 432.87 mV vs. Ag/AgCl. The difference in immersion potential between the sandblasted sample without a protective coating and the sample covered with paint to which kreutzonit particles were introduced was ΔE = 320.1 mV vs. Ag/AgCl, while at the end of the immersion period, the potential difference was ΔE = 854.99 mV vs. Ag/AgCl.

From Figure 6, it can be seen that all of the samples’ studied surfaces showed distinct variations in electrochemical potential behavior, indicating varied rates of anticorrosion protection depending on the treatment applied to their surfaces. Out of all of the surfaces studied, those coated with epoxy primer and then polyurethane paint to which kreutzonit particles had been added demonstrated the best anticorrosion behavior due to the formation of a protective oxide film on the surface of the material which, over time, protected the material from the aggressive action of natural seawater.

#### 3.4.2. Evolution of R_p_ and V_corr_ During Immersion Time

**Figure 7:**
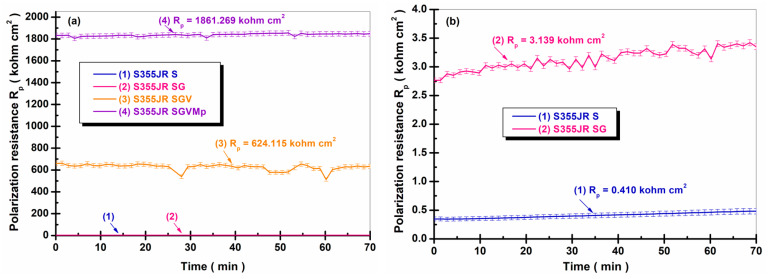
Variation in polarization resistance (R_p_) 1 h after immersion in natural seawater for (1) S355JR S, (2) S355JR SG, (3) S355JR SGV, (4) and S355JR SGVMp; (**a**) overview of the entire analyzed R_p_ domain and (**b**) zoomed-in version of the region with low R_p_ values.

Polarization resistance (R_p_) measurements were used to determine the protective capacity of the coatings on the surface of S355JR steel, since the R_p_ values recorded were inversely proportional to the corrosion current. The bias resistance is defined as the resistance of the sample to oxidation during the application of an external potential. The corrosion rate is directly related to R_p_ and can be calculated using the Stern–Geary equation (Equations (1) and (2)) [36,49,50]. The advantage of the R_p_ technique is that the measurement is simple and fast (response time is only a few minutes) and the corrosion rate is evaluated almost instantaneously. Figure 7 shows the variation in polarization resistance (R_p_) 1 h after immersion in natural seawater for the studied surfaces.

From the analysis of Figure 7, an increase in the values of the polarization resistance can be observed with the application of anticorrosion protection layers compared to the S355JR steel sandblasted with Al_2_O_3_. It is well known in the specialized literature that the polarization resistance is inversely proportional to the corrosion rate; in other words, the higher the polarization resistance of a studied material, the lower the corrosion rate [36]. In our case, the effectiveness of the anticorrosion protection layer to which kreutzonit particles were added was proven. S355JR S had an average polarization resistance value of R_p_ = 0.410 ± 0.034 kohm cm^2^, while at the S355JR SG surface, an increase in polarization resistance was observed to a value of R_p_ = 3.139 ± 0.169 kohm cm^2^. This value demonstrates that after one hour of immersing the samples in natural seawater, the resistance of the sandblasted steel over which a layer of zinc-enriched epoxy primer was applied was approximately 8 times higher than the steel sandblasted with Al_2_O_3_ without a protective coating.

For S355JR SGV, it was observed that it had a polarization resistance value of R_p_ = 624.115 ± 35.278 kohm cm^2^, a value that was approximately 188 times higher than S355JR SG and approximately 1522 higher than S355JR S.

The highest polarization resistance was obtained by S355JR SGVMp, which had a value of 1861.269 ± 23.351 kohm cm^2^.

**Figure 8:**
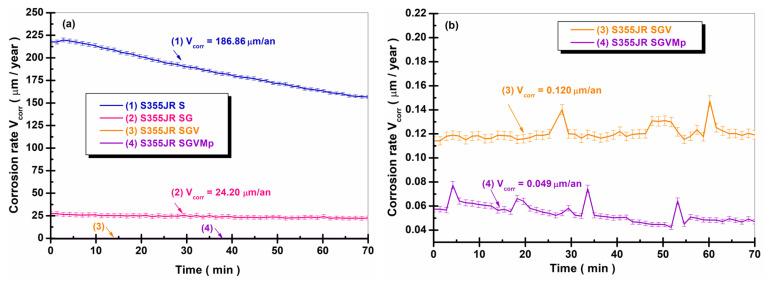
Variation in corrosion rate expressed as penetration rate (V_corr_), 1 h after immersion in natural seawater for (1) S355JR S, (2) S355JR SG, (3) S355JR SGV, and (4) S355JR SGVMp; (**a**) overview of the entire analyzed V_corr_ domain and (**b**) zoomed-in version of the region of low V_corr_ values.

Similar results were observed in the literature by Ramezanzadeh B. and colleagues [22], who studied the effect of adding ZnO microparticles and nanoparticles on the corrosion resistance of an epoxy–polyamide coating on hot-dipped galvanized steel for 120 days in a 3.5% NaCl solution. They demonstrated that the addition of ZnO microparticles and nanoparticles in the epoxy–polyamide coating improves the corrosion resistance of hot-dipped galvanized steel compared to the blank sample [22]. Also, Benea L. and collaborators [12] studied the effect of adding TiO_2_ nanoparticles in an epoxy coating on the corrosion resistance of an E32 steel. They demonstrated that by adding TiO_2_ nanoparticles to the epoxy primer, the polarization resistance of E32 steel increased compared to the same material without a protective coating. Corrosion rate expressed as penetration rate (presented in Figure 8) is the rate at which any metal deteriorates in a certain environment in a period of time.

The rate of deterioration depends on environmental conditions, as well as the type and condition of the reference metal. More data must be collected when calculating the corrosion rate of any material under study. The required data include (i) weight loss (the loss in weight of the metal during the reference period); (ii) density of the metal; (iii) the total area initially present; and (iv) the length of time considered. The data acquisition program calculates the results based on all the necessary information provided [51].

From the analysis of Figure 8, a decrease in the corrosion rate values can be observed with the application of anticorrosion protection layers compared to the S355JR S without a protective coating. S355JR S had an average value for corrosion rate V_corr_ = 186.86 ± 19.63 µm/year, while for the surface of S355JR SG, a decrease in the value of V_corr_ was observed to a value of 24.20 ± 1.32 µm/year.

This value demonstrates that after one hour of immersing the samples in natural seawater, the corrosion rate expressed as penetration rate of the sandblasted steel over which a layer of zinc-enriched epoxy primer was applied was approximately 8 times lower than the steel sandblasted with Al_2_O_3_ without a protective coating.

For S355JR SGV, it was observed that it had a corrosion rate of 0.120 ± 0.008 µm/year, a value approximately 200 times lower than S355JR SG and approximately 1550 smaller than S355JR S. The lowest corrosion rate was obtained by S355JR SGVMp, which had a value of 0.049 ± 0.002 µm/an. Table 5 shows the values obtained for the polarization resistance and corrosion rate of S355JR S, S355JR SG, S355JR SGV, and S355JR SGVMp immersed in natural seawater for 22 weeks.

From Table 5, it can be seen that at immersion, the S355JR S sample had an R_p_ value of 0.410 ± 0.034 kohm cm^2^. After 2 weeks of immersion, this value increased to 1.240 ± 0.026 kohm cm^2^, indicating that a protective oxide layer had formed on the surface of the studied material. From time T3 to T9, the values continue to decrease, indicating that the surface of the studied material was degrading at an accelerated rate.

After 16 weeks, the polarization resistance and corrosion rate values stabilized at slightly fluctuating values, indicating that the material had reached a dynamic equilibrium where the corrosion rate remained constant. For S355JR SG, it was observed that the R_p_ values were higher compared to the S355JR S surface, indicating that the epoxy primer applied over the sandblasted S355JR steel offered better anticorrosion protection than the studied material without coating protection.

For the sandblasted coated steel over which the zinc-enriched epoxy primer was applied, it can be observed that in the first 4 weeks, there was an increase in R_p_ values; then, in weeks 6 and 8, there was a decrease in R_p_ values. In week 10, there was a slight increase in R_p_ values compared to week 8; after this period of immersion, there was a decrease in values with slight fluctuations towards the end of the monitoring period.

For S355JR SGV, it was observed that the R_p_ values were much higher compared to the samples S355JR S and S355JR SG, thus confirming that this coating system for naval steel (zinc-enriched epoxy primer and polyurethane paint) provided the best protection against corrosion [40].

For S355JR SGV, an increase in R_p_ values was observed for 6 weeks, after which there was a stabilization of the values in weeks 8 and 10, followed by a slight increase in R_p_ values in weeks 12 and 14, stabilizing towards the end of the monitoring period.

In the case of S355JR SGVMp, higher R_p_ values were observed compared with S355JR S_,_ S355JR SG, and S355JR SGV. This behavior indicated that the coating to which kreutzonit particles were added provided the most effective protection against corrosion processes in the natural marine environment. The samples with the addition of kreutzonit particles showed R_p_ values 2 times higher than those of S355JR SGV, providing 2 times greater protection compared to the same surface without the addition of kreutzonit microparticles.

### 3.5. Structural FTIR Analysis Before and After Corrosion

**Figure 9:**
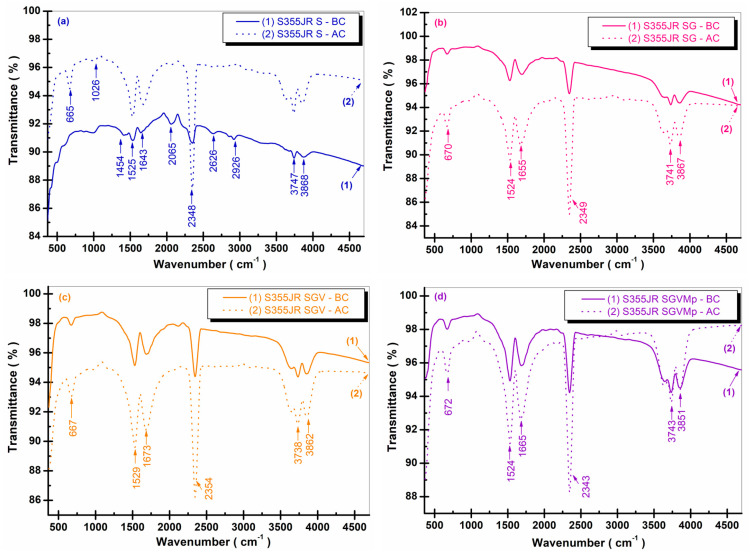
FTIR spectra for (**a**) S355JR S; (**b**) S355JR SG; (**c**) S355JR SGV; and (**d**) S355JR SGVMp. Lines labeled (1) are the spectra before corrosion and lines labeled (2) are the spectra after corrosion in natural seawater.

The structural characteristics of coated and uncoated S355JR steel samples before and after corrosion were studied using FTIR spectroscopy and are presented in Figure 9.

Analyzing the spectra (Figure 9a), it can be observed that there are evident absorption peaks in the spectrum of S355JR S after corrosion (curve 2) compared to the same sample before corrosion (curve 1).

This sample exhibits characteristic absorption bands at 665, 1026, 1454, 1525, 1643, 2065, 2348, 2626, 2926, 3747, and 3868 cm^−^^1^. The band observed at 665 cm^−^^1^, which appears only in the sample after corrosion (curve 2), is attributed to the Fe-O bond [52,53,54,55].

The band observed at 1026 cm^−^^1^ also appears only in the sample after corrosion (curve 2) and is attributed to lepidocrocite [53,56]. The bands at 1454 cm^−^^1^ and 1525 cm^−^^1^ are attributed to oxyhydroxides [53]. The band at 1643 cm^−^^1^ is attributed to the C-O bond, while the bands at 3747 cm^−^^1^ and 3868 cm^−^^1^ are attributed to OH bonds [53]. The band at 2348 cm^−^^1^ is attributed to the C=C bond, and the band at 2626 cm^−^^1^ is attributed to the C-H bond (likely due to the presence of organic compounds or organic microbial fractions present in seawater); the band at 2926 cm^−^^1^ is attributed to green rust, an intermediate complex of Fe(OH)_2_, formed due to oxidation in the presence of air.

However, it is very unstable and undergoes conversion into other morphologies, such as goethite, magnetite, or akaganeite, through subsequent oxidation processes [53].

It is worth noting that the appearance of corrosion products is significantly more pronounced in the S355JR S sample after corrosion (curve 2), as also highlighted by SEM and XRD analyses. The increase in transmittance in the sample after corrosion compared to the sample before corrosion may be due to changes in the crystalline structure of the material and the accumulation of corrosion products (unevenly distributed on the surface), which absorb light energy in different proportions, in a way that increases transmittance.

From Figure 9b, it can be observed that in the case of the S355JR SG before corrosion, the transmittance is lower compared to the S355JR S sample after corrosion (Figure 9a).

This behavior is possibly due to the protective coating and the corrosion products formed on the surface of the material, which affect its ability to allow light to pass through.

In the S355JR SG sample before and after corrosion, the absorption bands shifted slightly to higher or lower values and are fewer compared to the S355JR S sample.

The S355JR SG sample exhibits characteristic absorption bands at 670, 1524, 1655, 2349, 3741, and 3867 cm^−^^1^. In the case of the S355JR steel blasted with Al_2_O_3_, the band observed at 665 cm^−^^1^, which appears only in the sample after corrosion (curve 2), is attributed to the Fe-O bond (magnetite) [52,53,54,55]. It can be seen that in the S355JR SG sample, before and after corrosion, the Fe-O band shifts to higher values compared to the blasted sample without a protective coating and appears at 670 cm^−^^1^ in the samples before and after corrosion. The appearance of the characteristic absorption bands of iron oxides in the S355JR SG sample before corrosion is due to the iron oxide pigments used to provide resistance to discoloration and degradation under various light and environmental exposure conditions.

As with the sample analyzed previously in Figure 9b, for the S355JR SGV sample before and after corrosion (Figure 9c), the same absorption bands are observed, which have shifted slightly to higher or lower values. The S355JR SGV sample exhibits characteristic absorption bands at 667, 1529, 1673, 2354, 3738, and 3862 cm^−^^1^. The appearance of the characteristic absorption bands of iron oxides in the S355JR SGV sample before corrosion is due to the iron oxide pigments used to provide resistance to discoloration and degradation under various light and environmental exposure conditions.

Figure 9d shows absorption bands that have shifted slightly to lower or higher values compared to the other analyzed samples, with characteristic bands at 672, 1524, 1665, 2343, 3743, and 3851 cm^−^^1^. While the S355JR S sample before and after corrosion showed the most characteristic iron oxide bonds, in the investigated steel surfaces with different coatings, the disappearance of absorption peaks corresponding to the 1026 and 1454 cm^−^^1^ bands was observed.

### 3.6. SEM-EDX Analysis Before and After Corrosion

**Figure 10:**
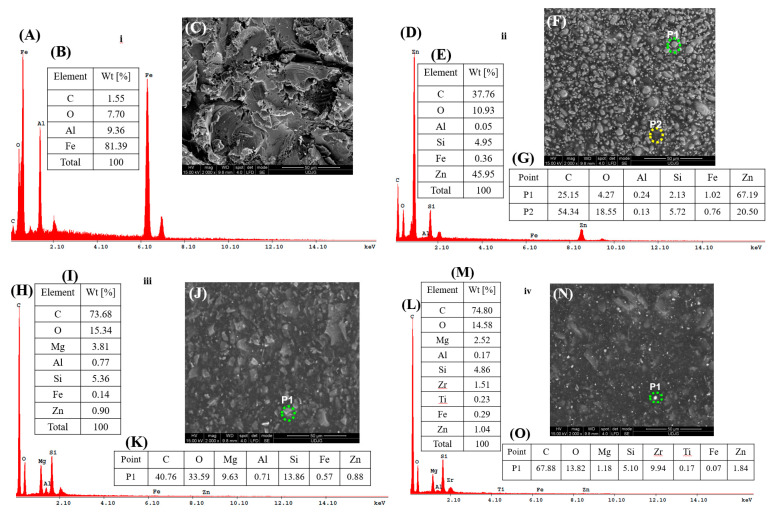
SEM-EDX analysis before corrosion for (**i**) S355JR S, (**ii**) S355JR SG, (**iii**) S355JR SGV, and (**iv**) S355JR SGVMp. (**A**,**D**,**H**,**L**) EDX spectrum, (**B**,**E**,**I**,**M**) general analysis over the entire surface of the micrograph, (**C**,**F**,**J**,**N**) SEM images, and (**G**,**K**,**O**) point analyses.

Figure 10 presents the SEM-EDX morphology before corrosion for the studied surfaces.

From Figure 10i, it can be observed that the surface of the S355JR S sample before corrosion is rough and exhibits many deep asperities resulting from the blasting process.

The EDX analysis for the same sample shows that, in addition to the elements Fe, O, and C, aluminum is also present with a mass percentage of 9.36 wt%. The presence of aluminum is due to the blasting sand used (Al_2_O_3_).

For the S355JR SG sample (Figure 10ii), the SEM morphology before corrosion shows a rough surface with many globular formations, which, upon point analysis, are found to be due to the presence of zinc in the epoxy primer used.

From the general analysis of the entire surface of the micrograph, it can be observed that, in addition to the S355JR S sample, the S355JR SG sample contains the elements Si and Zn in proportions of 4.95 wt% and 45.95 wt%, respectively.

Some researchers affirm that the use of silica in paint and coating compositions brings multiple advantages, including improved adhesion, maintenance of constant quality and performance, and reduced environmental impact [57]. Through its contribution to the development of durable and resistant coatings, silicon dioxide is becoming an essential component of modern paint and coating formulations [57].

From Figure 10iii, a different morphology was observed for the S355JR SGV sample compared to the S355JR SG sample. While Figure 10ii shows a morphology with globular formations due to the presence of zinc in the composition of the epoxy primer used, the S355JR SGV sample exhibits the presence of flakes, which, based on point analysis, can be attributed to the presence of MgO in the composition of the polyurethane paint used.

Stephen O’Driscoll [58] states in his doctoral work that the primary purpose of using MgO as a pigment in paints is to enhance the brightness, performance, and opacity of coatings, as well as for economic reasons, since MgO is a product that is much less expensive than TiO_2_ [58]. Compared to the samples presented in Figure 10i,ii, Figure 10iii shows the presence of Mg in addition to the elements in the other analyzed samples, as well as an increase in the element C and a decrease in the element Fe.

In the uncoated blasted sample, the Fe element had a mass percentage of 81.39 wt% while in the sample coated with primer, Fe decreased to a mass percentage of 0.36 wt%.

In the sample blasted and coated with primer and paint, Fe decreased even further to a mass percentage of 0.14 wt%.

While a decrease in the Fe element was observed in the coated samples, the carbon content increased with the number of coatings applied to the sample. In the case of the S355JR S sample, the carbon element was present at a percentage of 1.55 wt%.

On the surface coated with epoxy primer, the carbon content increased to a value of 37.76% wt. For the sample coated with primer and paint, the carbon content rose further to 73.68% wt. while for the surface coated with primer and paint to which kreutzonit microparticles had been dispersed, the carbon value reached 74.80 wt%. The increase in the carbon element was primarily due to the chemical structure of the polymeric coatings. In the case of the blasted steel, the increase in carbon content compared to the C content provided by the supplier Moiron in the certificate of conformity may be attributed to impurities on the sample’s surface resulting from the blasting process.

For the S355JR SGVMp sample, a surface morphology similar to that presented in Figure 10iii was observed. However, what was additionally noted was the presence of kreutzonit particles, which were uniformly distributed across the surface of the sample. The presence of Zr as well as Ti was confirmed both through point analysis and from the general elemental analysis of this sample.

The presence of these elements was observed only in this specific sample due to the presence of ZrO_2_ and TiO_2_ oxides found in the kreutzonit powder, according to the chemical composition provided by the supplier.

**Figure 11:**
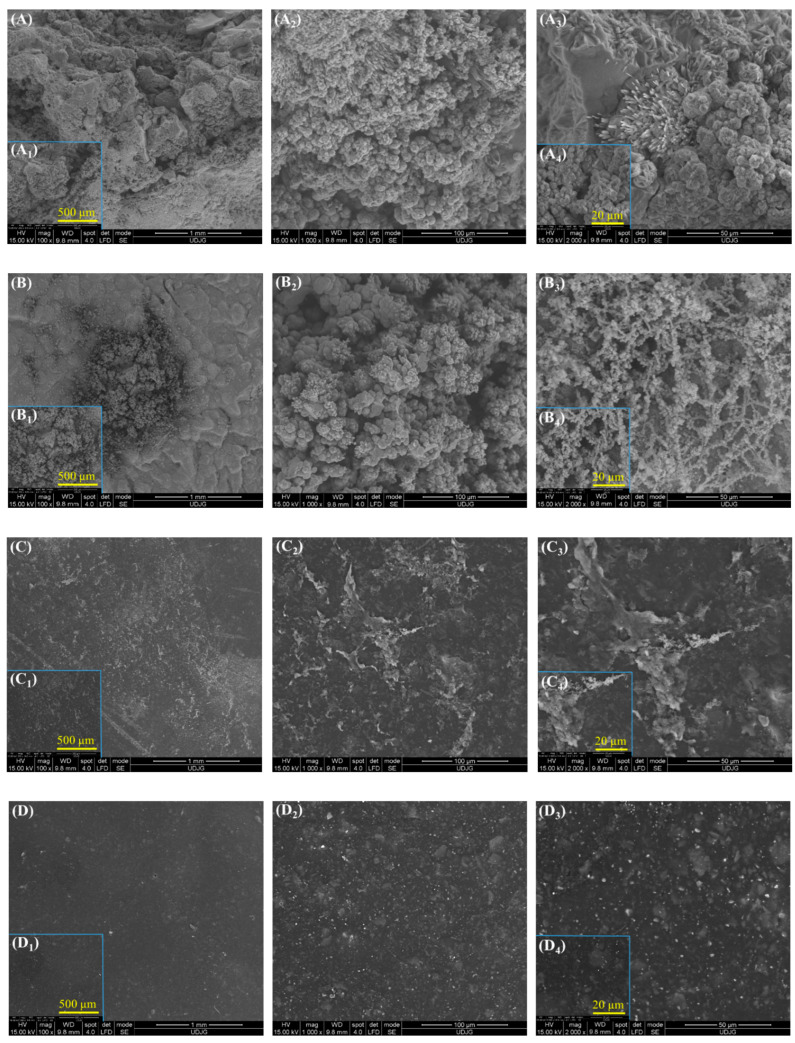
The SEM morphology after corrosion at different magnifications for (**A**) S355JR S, (**B**) S355JR SG, (**C**) S355JR SGV, and (**D**) S355JR SGVMp. (**A**–**D**) at 100×; (**A_1_**–**D_1_**) at 250×; (**A_2_**–**D_2_**) at 1000×; (**A_3_**–**D_3_**) at 2000×; and (**A_4_**–**D_4_**) at 5000×.

Figure 11 presents the SEM morphology after immersing the studied samples for 22 weeks in natural seawater. While in Figure 10, before corrosion, it is evident that the surfaces do not show corrosion products, after the corrosion process, different morphological structures associated with the corrosion products formed on the surface of the samples are observed, which are also confirmed by XRD, FTIR, and electrochemical analyses.

In Figure 11A, it can be observed that the S355JR S sample without a protective coating was the most affected by corrosion products, with its entire surface covered by these formed products. In Figure 11(A_2_–A_4_), the appearance of lamellar needle-like morphological formations and flower petal-shaped formations can be observed. According to studies from the specialized literature, these formations are attributed to the corrosion product γ-FeO(OH)—lepidocrocite [53,59,60,61,62,63,64,65].

Additionally, in the same sample, the appearance of flat, dark surface morphologies, attributed to magnetite, can be observed, as well as globular morphological formations attributed to the formation of the corrosion product α-FeO(OH) [53,59,60].

These morphological formations were also confirmed by XRD analysis. It is evident that the S355JR S sample suffered from generalized corrosion over the entire surface of the sample. In contrast, in the S355JR SG sample, pitting corrosion was observed, which did not affect the entire surface of the sample.

In Figure 11B, the appearance of globular morphologies as well as nest-like formations can be observed, attributed to α-FeO(OH) and γ-FeO(OH) [53,59,60,61,62,63,64,65]. In the S355JR SGV sample, a form of localized corrosion with a dense structure was observed, which, based on XRD analysis, can be identified as corrosion products (hematite and magnetite).

Morphology like the one in Figure 11(C–C4) has also been observed in the specialized literature by other authors who studied corrosion on a different type of material [64].

By conducting a comparative analysis of the morphologies presented in Figure 11, it was evident that the sample most affected by corrosion products was the S355JR S sample, and the least affected by corrosion products was the S355JR SGVMp sample.

**Figure 12:**
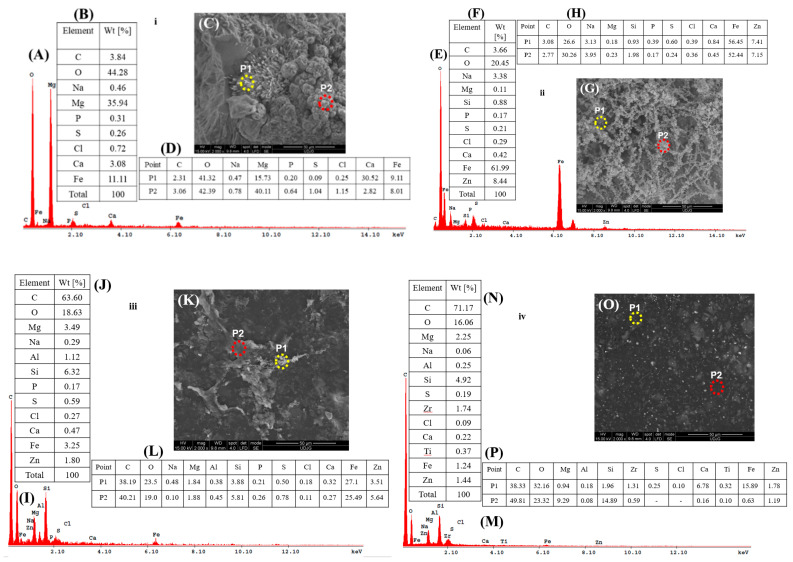
SEM-EDX analysis after corrosion for (**i**) S355JR S, (**ii**) S355JR SG, (**iii**) S355JR SGV, and (**iv**) S355JR SGVMp. (**A**,**E**,**I**,**M**) EDX spectrum, (**B**,**F**,**J**,**N**) general analysis of the entire surface of the SEM images, (**C**,**G**,**K**,**O**) SEM images, and (**D**,**H**,**L**,**P**) point-specific analyses.

To highlight the corrosion products formed on the surface of the samples shown in Figure 11, Figure 12 presents both general and point-specific elemental analysis of the studied samples after 22 weeks of corrosion testing in natural seawater.

In Figure 12, for the S355JR S sample, it can be observed that the general analysis of the entire surface shows an oxygen content of 44.28 wt%. Comparing this with the same sample before corrosion (Figure 10i(B)), the oxygen content increased by 36.58 wt% after the corrosion process, indicating the formation of oxides (corrosion products) on the surface of the S355JR S sample. It is evident that after corrosion, other elements such as Mg, Na, P, S, Cl, and Ca, which are present in natural seawater, appear. In Figure 12ii, there is also an observed increase in the oxygen content after the corrosion process for the S355JR SG sample. In this case, the increase in the oxygen element after corrosion compared to the same sample before corrosion is 9.52 wt%.

This behavior also indicates the formation of corrosion products on the surface of this sample, which is further confirmed by the XRD analyses. In terms of quantity, it is evident that the corrosion products on this sample are less prevalent compared to the sample without a protective anticorrosion coating.

For the S355JR SGV sample (Figure 12iii), the oxygen element value after corrosion was 18.63 wt%, while before corrosion, the value of the same element was 15.34 wt%. The O_2_ value after corrosion was 3.29 wt% higher than the oxygen value before the corrosion process.

The smallest difference in the increase in the O_2_ element was observed for the S355JR SGVMp sample (Figure 12iv), which had a value of 16.06 wt%, while before corrosion (Figure 10iv), it had a value of 14.58 wt%. The increase in the O_2_ element after corrosion for this sample was 1.48 wt%. This behavior confirms the effectiveness of the coating enhanced with kreutzonit particles.

### 3.7. XRD Analysis Before and After Corrosion

**Figure 13:**
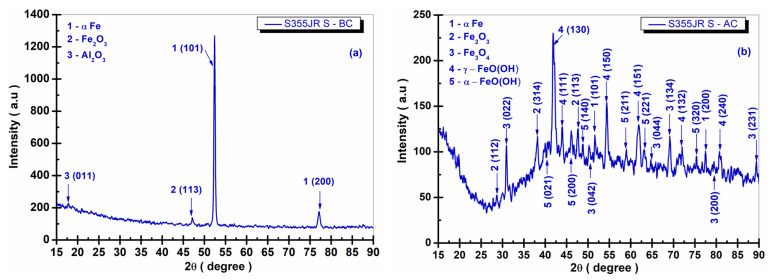
XRD patterns of S355JR S (**a**) before corrosion and (**b**) after corrosion in natural seawater.

**Figure 14:**
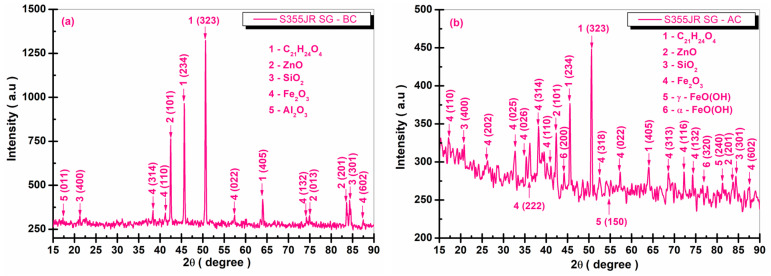
XRD patterns of S355JR SG (**a**) before corrosion and (**b**) after corrosion in natural seawater.

**Figure 15:**
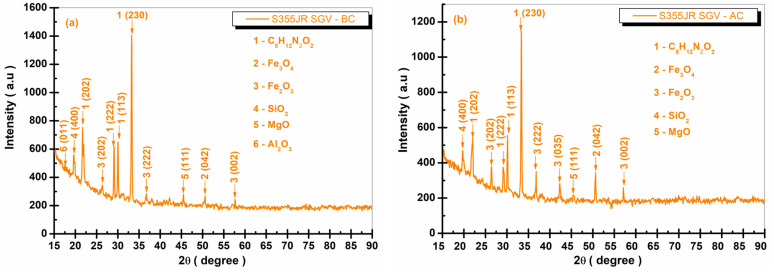
XRD patterns of S355JR SGV (**a**) before corrosion and (**b**) after corrosion in natural seawater.

**Figure 16:**
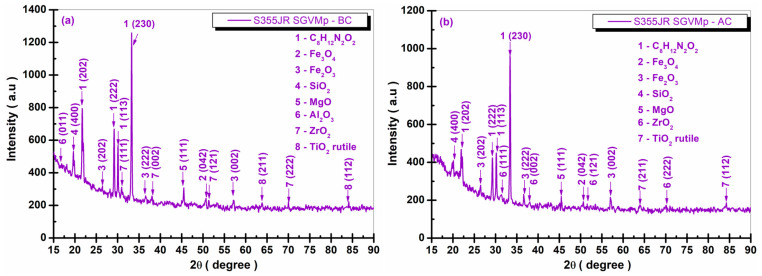
XRD patterns of S355JR SGVMp (**a**) before corrosion and (**b**) after corrosion in natural seawater.

Figure 13, Figure 14, Figure 15 and Figure 16 present the XRD diffraction patterns recorded for the S355JR S, S355JR SG, S355JR SGV, and S355JR SGVMp samples, before and after corrosion.

In Figure 13a, it can be observed that before corrosion, the S355JR S sample exhibited crystalline structural phases of αFe, Fe_2_O_3_, and Al_2_O_3_. The crystalline phase αFe was identified using the Crystallography Open Database (COD) 96-110-0109, which belongs to the cubic crystallization system, space group Im-3m(229), at the angles 2θ (52.35° and 77.19°) with the crystallographic planes (101 and 200). Additionally, the crystalline phase Fe_2_O_3_ was identified before corrosion using COD 96-210-8029, which belongs to the monoclinic crystallization system, space group C12/c1(15), at the angle 2θ (47.20°) with the crystallographic plane (113).

The crystalline phase Al_2_O_3_ was identified using COD 96-100-0443 at the angle 2θ (16.88°) with the crystallographic plane (011), belonging to the orthorhombic crystallization system, space group Pna21(33). While Figure 13a shows the presence of three peaks in the XRD spectrum before corrosion, after corrosion (Figure 13b), there was an increase in the number of peaks in the XRD spectrum compared to the sample before corrosion, indicating the formation of new crystalline phases as a result of the corrosion process.

The crystalline phases formed as a result of the corrosion process (Figure 13b) include αFe, Fe_2_O_3_, Fe_3_O_4_, γ-FeO(OH), and α-FeO(OH). In addition to the sample studied before corrosion, the crystalline phase Fe_2_O_3_ was identified after corrosion with the crystallographic planes (112 and 314) at the angles 2θ (28.71° and 38.22°), using the same COD and belonging to the same crystallization system and space group.

For the crystalline phase Fe_3_O_4_, identified using COD 96-900-2027, which belongs to the orthorhombic crystallization system, space group Pbcm(57), the crystallographic planes (022, 042, 134, 200, and 231) were identified at the angles 2θ (31.14°, 50.15°, 69.70°, 79.39°, and 89.77°).

It was identified that after immersing the S355JR S sample for 22 weeks in natural seawater, the crystalline phase γ-FeO(OH) was formed, present at the angles 2θ (42.12°, 43.79°, 54.32°, 62.12°, 71.89°, and 81.24°) with the crystallographic planes (130, 111, 150, 151, 132, and 240). This phase was identified using COD 96-901-7383 and belongs to the orthorhombic crystallization system, space group Cmc21(36). Additionally, the crystalline phase α-FeO(OH) was also formed, identified with COD 96-900-3079, belonging to the orthorhombic crystallization system, space group Pbnm(62), with the crystallographic planes (200, 021, 140, 211, 221, and 320) at the angles 2θ (46.06°, 40.53°, 48.41°, 59.06°, 63.20°, and 75.26°).

The formation of these crystallographic phases has been observed in the specialized literature by other authors who studied various types of carbon steels in synthetic or natural marine environments [7,53,66].

From these results, it is evident that the S355JR S sample without a protective layer is susceptible to accelerated corrosion due to the direct exposure of the material to the corrosive environment. The absence of a physical or chemical barrier between the surface of the sample and the corrosion factors allows for the penetration and direct interaction of corrosive substances present in natural seawater with the material, leading to faster and more extensive degradation [11,12,22].

The material is thus exposed to oxidation and degradation processes, as well as to the formation of corrosive products, which can lead to the loss of mechanical and functional properties of the material. The absence of a protective layer is a crucial factor in amplifying and accelerating the corrosion process, resulting in significant damage to the material over a shorter period compared to materials with anticorrosion protective coatings [11,12,22]

In Figure 14a, it can be observed that before corrosion, the S355JR SG sample exhibited crystalline structural phases of C_21_H_24_O_4_, ZnO, SiO_2_, Fe_2_O_3_, and Al_2_O_3_. The crystalline phase C_21_H_24_O_4_ was identified using COD 96-223-3718, which belongs to the orthorhombic crystallization system, space group P212121(19), at the angles 2θ (47.73°, 50.58°, and 61.20°) with the crystallographic planes (234, 323, and 405).

The crystalline phase ZnO was identified using COD 96-210-7060, which belongs to the hexagonal crystallization system, space group P63mc(186), at the angles 2θ (42.42°, 74.77°, and 83.94°) with the crystallographic planes (101, 013, and 201). The crystalline phase SiO_2_ was identified using COD 96-153-0729, which belongs to the cubic crystallization system, space group Fd-3m(227), at the angles 2θ (21.21° and 84.63°) with the crystallographic planes (400 and 301).

The crystalline phase Fe_2_O_3_ was also identified, belonging to the monoclinic crystallization system, space group C12/c1(15), at the angles 2θ (38.22°, 41.21°, 57.37°, 73.16°, and 87.26°) with the crystallographic planes (314, 110, 022, 132, and 602), identified using COD 96-210-8029. The phase Al_2_O_3_ was identified with COD 96-100-0443 at the angle 2θ (16.88°) with the crystallographic plane (011), which belongs to the orthorhombic crystallization system, space group Pna21(33).

The presence of iron(III) oxide before corrosion may also be attributed to the presence of iron oxide pigments used in the epoxy primer. After corrosion, in Figure 14b, a decrease in the intensity of the characteristic peaks of the crystalline phases C_21_H_24_O_4_, ZnO, and SiO_2_ was observed, along with an increase in the peaks characteristic of iron oxide (confirming the formation of corrosion products on the surface of the sample). In addition to the peaks identified before corrosion, attributed to Fe_2_O_3_, new peaks appear after corrosion at the angles 2θ (17.40°, 26.28°, 32.71°, 35.30°, 36.39°, 52.60°, 68.56°, and 72.34°) with the crystallographic planes (110, 202, 025, 026, 222, 318, 313, and 116), belonging to the space group P41212(92) of the tetragonal crystallization system, identified using COD 96-152-8613.

After corrosion, the crystallographic phases belonging to γ-FeO(OH) appear, present at the angles 2θ (54.32° and 81.24°) with the crystallographic planes (150 and 240), identified using COD 96-901-7383, which belongs to the orthorhombic crystallization system, space group Cmc21(36). Additionally, the crystalline phase α-FeO(OH) was identified using COD 96-900-3079, which belongs to the orthorhombic crystallization system, space group Pbnm(62), with the crystallographic planes (200 and 320) at the angles 2θ (46.06° and 75.26°).

By comparing the uncoated sample after corrosion with the S355JR SG sample after corrosion, it is evident that the characteristic peaks of the corrosion products are less intense and fewer in number compared to those on the uncoated sample. This behavior demonstrates that the primer layer applied to S355JR steel significantly reduces the occurrency of corrosion products [67].

From Figure 15a, the following crystallographic phases are observed: C_8_H_12_N_2_O_2_, Fe_3_O_4_, Fe_2_O_3_, SiO_2_, MgO, and Al_2_O_3_. C_8_H_12_N_2_O_2_ is a chemical compound used in the production of aliphatic polyurethane coatings and has been identified with COD 96-150-6880, showing representative peaks at the angles 2θ (21.97°, 29.09°, 29.85°, and 33.03°) with the crystallographic planes (202, 222, 113, and 230), belonging to the monoclinic crystallization system, space group P121/c1(14). The crystalline phase Fe_3_O_4_ has a representative peak at the angle 2θ (50.15°) with the crystallographic plane (042), belonging to the orthorhombic crystallization system, space group Pbcm(57), identified using COD 96-900-2027. Fe_2_O_3_, identified with COD 96-152-8613, shows representative peaks at the angles 2θ (26.28° and 36.39°) with the crystallographic planes (202 and 222), belonging to the tetragonal crystallization system, space group P41212(92).

It has also been identified that the crystalline phase Fe_2_O_3_, belonging to the monoclinic crystallization system, space group C12/c1(15), is identified with COD 96-210-8029 at the angle 2θ (57.27°) with the crystallographic plane (002).

The presence of iron oxides before corrosion may be due to the presence of iron oxide pigments used in the paint to give it a black color. The crystalline phase SiO_2_, identified with COD 96-153-0729, belongs to the cubic crystallization system, space group Fd-3m (227), at an angle of 2θ (21.21°) with the crystallographic plane (400). The crystalline phase MgO, which belongs to the cubic crystallization system, space group Fm-3m (225), is identified with COD 96-901-3203 at an angle of 2θ (45.03°) with the crystallographic plane (111).

The phase Al_2_O_3_ has been identified with COD 96-100-0443 at an angle of 2θ (16.88°) with the crystallographic plane (011), belonging to the orthorhombic crystallization system, space group Pna21(33).

After corrosion, in Figure 15b, a decrease in intensity of the characteristic peaks of the crystalline phase C_8_H_12_N_2_O_2_ was observed, along with a slight increase in the peaks characteristic of iron oxide (confirming the presence of corrosion products on the surface of the sample). In addition to the peaks identified before corrosion, attributed to Fe_2_O_3_, a peak appears after corrosion at an angle of 2θ (43.21°) with the crystallographic plane (035) belonging to the space group P41212(92) of the tetragonal crystal system, identified with COD 96-152-8613.

Making a comparison between the uncoated sample after corrosion, S355JR steel blasted and coated with epoxy primer after corrosion, and S355JR steel blasted and coated with epoxy primer and polyurethane paint, it is evident that the characteristic peaks of corrosion products at the sample S355JR SGV are lower in intensity and fewer in number compared to the samples presented in Figure 13b and Figure 14b. Thus, it is demonstrated that the combination of the epoxy primer layer and polyurethane paint offers superior corrosion protection compared to the coating with the epoxy primer, without the polyurethane paint [68,69].

In addition to the phases identified in the S355JR SGV sample, the presence of the crystallographic phases ZrO_2_ and TiO_2_, found in the chemical composition of the micrometric powder of kreutzonite, was observed at the S355JR SGVMp sample.

The crystalline phase ZrO_2_ was identified with COD 96-152-2144, showing representative peaks at the angles 2θ (31.01°, 38.08°, 51.42°, and 69.82°) corresponding to the crystallographic planes (111, 002, 121, and 222) belonging to the monoclinic crystal system, space group P121/c1(14). The TiO_2_ rutile phase was identified with COD 96-900-4142 at the angles 2θ (63.79° and 83.71°) with the crystallographic planes (211 and 112) belonging to the tetragonal crystal system, space group P42/mnm(136). Regarding the presence of iron oxides, compared to the sample presented in Figure 15b, Figure 16b shows the absence of the peak at the angle 2θ (43.21°) corresponding to the crystallographic plane (035).

**Figure 17:**
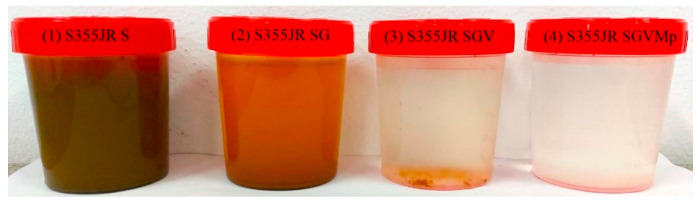
The aspect of the electrolyte after the corrosion process (22 weeks) for (1) S355JR S, (2) S355JR SG, (3) S355JR SGV, and (4) S355JR SGVMp.

Moreover, the increase in intensity of the peaks attributed to iron oxides after corrosion in the S355JR SGVMp sample is practically imperceptible. These results are consistent with the electrochemical tests conducted as well as the qualitative physical analysis (the color of natural seawater) after 22 weeks of immersion of the samples. Figure 17 shows the appearance of the natural seawater used as an electrolyte for the electrochemical tests after the prolonged immersion of the investigated steel surfaces.

After the corrosion process, the appearance of reddish-brown sediment flakes is clearly observed, which have gradually settled at the bottom of the container over time in the samples that underwent corrosion processes. This image confirms the effectiveness of the protective layer, to which kreutzonit particles were added; this coating provided the most effective protection against corrosion processes in the natural marine environment compared to the other investigated steel surfaces studied in this work.

## 4. Conclusions

In this study, the corrosion behavior and protective performance of coatings applied to S355JR structural steel were evaluated over a period of 22 weeks of exposure to natural seawater using advanced analytical techniques and electrochemical tests. The main conclusions derived from the experimental results are as follows:

Electrochemical analysis indicated that the layer consisting of epoxy primer and polyurethane paint, with the addition of kreutzonit particles, provided the most effective protection against corrosion processes in the natural marine environment.

Morphological analysis demonstrated that surfaces blasted with Al_2_O_3_ exhibited significant asperities conducive to corrosion initiation, whereas coated surfaces formed uniform and homogeneous layers, reducing surface roughness and enhancing anticorrosive performance. After immersion analyses revealed extensive formation of corrosion products (lepidocrocite, magnetite, and iron hydroxides) on the uncoated sample, whereas the coating with added kreutzonit particles exhibited minimal degradation. EDX analysis confirmed a significant increase in oxygen content on the uncoated surface after immersion in natural seawater, whereas the coating with kreutzonit particles showed a reduced increase in oxygen content, highlighting the efficiency of this layer in preventing oxygen penetration and the formation of corrosion products.

FTIR spectroscopy revealed a significant attenuation of Fe-O absorption bands in the coated samples, particularly in those with kreutzonit particles, while XRD patterns confirmed a reduction in the formation of crystalline corrosion phases (Fe_2_O_3_, Fe_3_O_4_, γ-FeO(OH), and α-FeO(OH)) compared to the uncoated samples.

Although a decrease in microhardness was observed due to the polymeric nature of the protective coatings compared to the uncoated samples, the addition of kreutzonit particles increased hardness values compared to coatings without kreutzonit particles, indicating improved mechanical integrity.

Measurements of surface roughness and contact angle demonstrated an inverse correlation; uncoated samples exhibited high roughness and reduced hydrophobicity, while coatings with added kreutzonit particles showed the lowest roughness and the highest contact angle, emphasizing their superior barrier properties.

These results confirm that the addition of kreutzonit particles to epoxy-polyurethane coatings significantly improves the performance of these coatings in preventing corrosion of steel in natural seawater.

Future studies will continue to monitor performance over longer periods of time (years) and under conditions of temperature variation, as well as investigate the influence of natural corrosion inhibitors on the coatings developed and tested in this study.

## Figures and Tables

**Table 1:** Chemical composition of S355JR steel [%].

C	Si	Mn	P	S	N	Cu	Fe
0.24	0.55	1.60	0.035	0.035	0.012	0.55	Balance

**Table 2:** The physical and chemical properties of kreutzonit powder.

Chemical Properties	
ZrO_2_	64–65.5%
SiO_2_	33–34%
Fe_2_O_3_	0.10%
TiO_2_	0.15%
**Physical properties**	
Specific weight	4.6 g/cm^3^
Melting point	2200 °C
Mohs hardness	7.5
Particle size	5 µm

**Table 3:** Abbreviation adopted in this study.

Title	Abbreviation
S355JR steel blasted with Al_2_O_3_	S355JR S
S355JR steel blasted and coated with epoxy primer enriched with zinc	S355JR SG
S355JR steel blasted and coated with epoxy primer and polyurethane paint	S355JR SGV
S355JR steel blasted and subsequently coated with epoxy primer and then polyurethane paint to which kreutzonit particles had been added	S355JR SGVMp

**Table 4:** Physical–chemical characteristics of natural seawater.

pH	Electrical Conductivity [mS/cm]	Salinity [g/L]	TDS [ppt]
8.13	23.8	15.68	16.23

**Table 5:** The values of polarization resistance and corrosion rate expressed as penetration rate for investigated steel surfaces obtained during 22-week immersion in natural seawater.

Surface Studied	S355JR S		S355JR SG		S355JR SGV		S355JR SGVMp	
Times Studied	R_p_ [kohm cm^2^]	V_corr_ [µm/an]	R_p_ [kohm cm^2^]	V_corr_ [µm/an]	R_p_ [kohm cm^2^]	V_corr_ [µm/an]	R_p_ [kohm cm^2^]	V_corr_ [µm/an]
T1—1 h after immersion	0.410 ± 0.034	186.86 ± 19.63	3.139 ± 0.169	24.20 ± 1.32	624.115 ± 35.278	0.120 ± 0.008	1861.269 ± 23.351	0.0490 ± 0.002
T2—after 2 weeks	1.240 ± 0.026	60.941 ± 0.132	30.770 ± 0.120	2.458 ± 0.068	1221.80 ± 70.011	0.0745 ± 0.001	2445.15 ± 33.735	0.0328 ± 0.003
T3—after 4 weeks	1.178 ± 0.016	64.702 ± 0.567	31.569 ± 0.666	2.395 ± 0.050	1445.15 ± 34.100	0.0729 ± 0.006	2621.17 ± 25.127	0.0303 ± 0.002
T4—after 6 weeks	0.959 ± 0.108	79.706 ± 0.669	29.646 ± 0.750	2.550 ± 0.064	1728.46 ± 42.039	0.0454 ± 0.004	2989.09 ± 43.668	0.0241 ± 0.001
T5—after 8 weeks	0.158 ± 0.021	478.31 ± 28.453	28.281 ± 0.882	2.576 ± 0.034	1553.67 ± 48.932	0.0628 ± 0.002	3245.97 ± 76.196	0.0204 ± 0.003
T6—after 10 weeks	0.134 ± 0.009	561.26 ± 17.253	29.336 ± 0.019	2.563 ± 0.089	1550.28 ± 64.155	0.0630 ± 0.002	3305.42 ± 59.862	0.0185 0.012
T7—after 12 weeks	0.125 ± 0.014	604.45 ± 54.741	22.242 ± 0.329	3.398 ± 0.104	1628.54 ± 52.483	0.0546 ± 0.021	2950.28 ± 39.891	0.0252 ± 0.067
T8—after 14 weeks	0.112 ± 0.018	671.12 ± 60.073	12.286 ± 0.422	6.175 ± 0.832	1641.79 ± 48.746	0.0542 ± 0.007	2935.41 ± 41.743	0.0257 ± 0.002
T9—after 16 weeks	0.075 ± 0.005	1342.01 ± 81.439	13.261 ± 1.219	5.800 ± 0.742	1587.90 ± 37.958	0.0608 ± 0.004	2938.74 ± 34.285	0.0255 ± 0.003
T10—after 18 weeks	0.119 ± 0.010	631.60 ± 58.437	11.563 ± 1.162	6.538 ± 0.917	1528.82 ± 42.546	0.0664 ± 0.002	2943.71 ± 46.231	0.0254 ± 0.008
T11—after 20 weeks	0.114 ± 0.007	658.76 ± 59.018	15.565 ± 2.138	4.856 ± 0.429	1523.25 ± 40.129	0.0671 ± 0.002	2918.61 ± 52.195	0.0261 ± 0.001
T12—after 22 weeks	0.109 ± 0.011	692.75 ± 62.498	14.383 ± 2.018	5.257 ± 0.379	1493.66 ± 39.055	0.0705 ± 0.003	2919.99 ± 48.932	0.0262 ± 0.004

## Data Availability

The original contributions presented in this study are included in the article. Further inquiries can be directed to the corresponding authors.
